# Weak self-supervised learning for seizure forecasting: a feasibility study

**DOI:** 10.1098/rsos.220374

**Published:** 2022-08-03

**Authors:** Yikai Yang, Nhan Duy Truong, Jason K. Eshraghian, Armin Nikpour, Omid Kavehei

**Affiliations:** ^1^ School of Biomedical Engineering, and the Australian Research Council Training Centre for Innovative BioEngineering, Faculty of EngineeringThe University of Sydney Nano Institute, Sydney, New South Wales 2006, Australia; ^2^ The University of Sydney Nano Institute, Sydney, New South Wales 2006, Australia; ^3^ Department of Electrical and Computer Engineering, University of California Santa Cruz, Santa Cruz, CA 95064, USA; ^4^ Faculty of Medicine and Health, Central Clinical School, The University of Sydney, Sydney, New South Wales 2006, Australia; ^5^ Comprehensive Epilepsy Service and Department of Neurology, Royal Prince Alfred Hospital, Camperdown, New South Wales 2050, Australia

**Keywords:** adaptive forecasting and self-learning model, epileptic seizure forecasting, neuromorphic neuromodulation, online learning

## Abstract

This paper proposes an artificial intelligence system that continuously improves over time at event prediction using initially unlabelled data by using self-supervised learning. Time-series data are inherently autocorrelated. By using a detection model to generate weak labels on the fly, which are concurrently used as targets to train a prediction model on a time-shifted input data stream, this autocorrelation can effectively be harnessed to reduce the burden of manual labelling. This is critical in medical patient monitoring, as it enables the development of personalized forecasting models without demanding the annotation of long sequences of physiological signal recordings. We perform a feasibility study on seizure prediction, which is identified as an ideal test case, as pre-ictal brainwaves are patient-specific, and tailoring models to individual patients is known to improve forecasting performance significantly. Our self-supervised approach is used to train individualized forecasting models for 10 patients, showing an average relative improvement in sensitivity by 14.30% and a reduction in false alarms by 19.61% in early seizure forecasting. This proof-of-concept on the feasibility of using a continuous stream of time-series neurophysiological data paves the way towards a low-power neuromorphic neuromodulation system.

## 1. Introduction

Machine forecasting of future events provides an opportunity to integrate preventative systems across a variety of domains. In healthcare, disease prevention is preferable over disease management for both better patient outcomes and for medical resource management. In general, the performance of supervised machine learning models is subject to the quantity and quality of training data [1], which poses a major challenge in healthcare where labelled data are often lacking, and generalization across patients can be difficult to achieve and quantify [2–5]. In many instances, labelled data are only available at the time of the event, or a brief period of time preceding the event [6,7]. This significantly limits the flexibility of forecasting models. The relative scarcity of labelled datasets for event prediction and forecasting results in the under-performance of machine learning models for the early detection of many tasks [8–10].

In this paper, we present a novel forecasting AI system called ‘AURA’: an Adaptive forecasting model trained with Unlabelled, Real-time data using internally generated Approximate labels on-the-fly. We assess the performance of the system in the context of physiological signal recording and stimulation, where a forecasting network is trained using unlabelled, real-time data that relies on a detection network to provide autonomously generated labels. This is a special case of semi-supervised learning [11,12], where the Bayes error of the fixed network (detection) is less than that of the adjustable network (forecasting), even if the latter were to be considered ‘perfectly’ trained. In general, this assumption holds true, as ‘telling the future’ is considerably more challenging than ‘telling the present’. Several principles are used in using AURA:

— *Detection outperforms prediction:* In many applications, the performance of a forecasting model degrades as prediction interval increases. Indicators and bio-markers of events characteristically strengthen as the onset of an event approaches closer in time. The proposed method exploits this fact and uses a detection system to label data which then trains the forecasting model. This is performed with the expectation that incorrectly labelled data by the detection system are unlikely to be correctly predicted by the forecasting system. So emphasis is placed on enabling the predictive system to converge towards the performance of the detector, rather than pushing towards a potentially unrealistic goal of 100% accuracy. Although the asymptotic error of prediction is not shown to converge to that of detection, we show practical examples that demonstrate it works almost as well.
— *Continuously learning systems can potentially avoid the consequences of data drift:* Online learning can suffer from catastrophic interference (or catastrophic forgetting), where the onset of new training data ‘overrides’ the latent representations of historical data in a neural network [13]. In certain cases, it may be that new data are more representative of present circumstances, so it is preferable to learn from more recent information. More precisely, time-series data are often non-stationary, and the statistical properties of the incoming data are likely to evolve. Online learning could train a system to adapt to changing patient conditions, or data drift, over time.
— *Patient-specific tuning:* While the above point focuses on ‘new’ data in the temporal sense, it also holds true for ‘new’ patients. A model tuned to an individual patient is likely to perform better for that particular individual over a model trained to generalize across a multitude of patients (as is typically the case for most machine learning models in use today). This observation holds true beyond medical diagnostics, for example, geography-specific weather forecasting. Historically, the cost of manually labelling individual patient data to designing patient-specific models has been prohibitively expensive for large-scale deployment. AURA overcomes this using the semi-supervised approach described in §2.5.

### 1.1. Novelty and significance

Designing microelectronic circuits and systems for medical implants and electroceuticals is challenging and bounded by several constraints, such as area dimensions, energy consumption, safety, and the need for continuous or very regular data telemetry [14]. While it is not difficult to find medical devices with different capacities of performing on-chip analogue or digital signal processing, active on-chip learning use in the neuromodulation or neuromonitoring domain is in its infancy [15].

**Figure 1.**
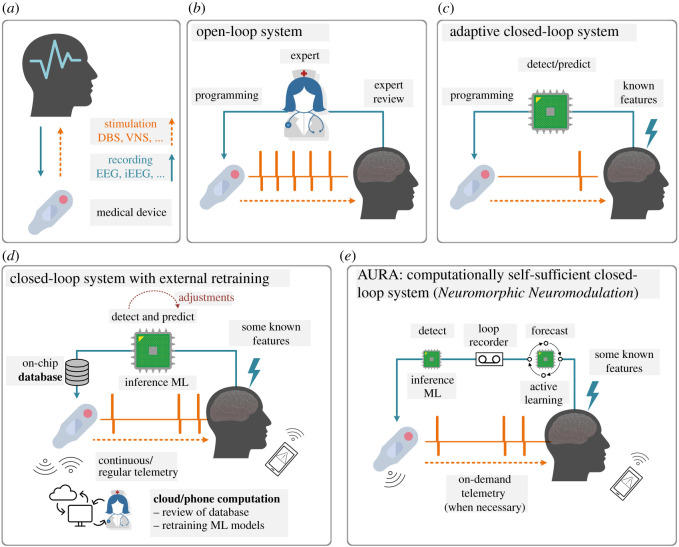
(*a*) Neuromonitoring (recording) and neuromodulation (stimulation) as part of a medical device and their different system types. While this method in theory can be used with any physiological signal, the most promising are electroencephalogram (EEG), intracranial electroencephalography (iEEG) and local field potentials (LFP), as part of neuromodulation systems such as vagus nerve stimulation (VNS) or deep brain stimulation (DBS). (*b*) An open-loop system with a human *expert in the loop* that engages occasionally in manual programming and parameter adjustment. (*c*) An adaptive closed-loop system with an automated bio-marker or feature detection and real-time signal processing that controls simulation. Examples include [9,16–22]. (*d*) An example of a *cloud or smartphone in the loop* system that allows for online adjustments of detection or prediction models, but retraining of the models using the stream of incoming data must be conducted on the cloud. These systems rely on either continuous or regular data telemetry of the data stored in the database. Examples include [23–32]. (*e*) AURA operates on the basis of a *neuromorphic neuromodulation system*, where the medical device hosts online training and active learning without any reliance on external computing resources. *Approximate learning* is used to train the active learning model, which pushes the performance of the probabilistic forecasting system towards the non-patient-specific inference machine-learning detection model. Once implemented on a low-power neuromorphic chip, system personalization is achieved via its computationally self-sufficient re-training of the forecasting model without the need for data telemetry to the outside world. There is an increasing body of evidence that highly customized low-power neuromorphic systems and models can deliver our vision [33,34].

Figure 1 demonstrates four general types of loops in medical devices, as applied to neurotechnologies (figure 1*a*). In an open-loop system (figure 1*b*), decision-making is reviewed by a trained human expert who may perform visits to reprogram the device. This indicates stimulation parameters such as amplitude, frequency and duty cycle are pre-identified and are indefinitely fixed for the duration of device operation, unless changed manually. This method lacks individualization, may require additional training to equip doctors with the programming skills to re-parameterize the device, exhausts the battery, and in the context of a closed-loop neurophysiological system, can result in a very high frequency of unnecessarily applied neurostimulation, or false positives [35]. An adaptive closed-loop system (figure 1*c*), however, can control its stimulation via a feedback loop using extracted bio-markers. Accepting a slightly higher risk of automation in feature extraction by reducing the significance of *expert-in-the-loop* intervention enables significant energy savings, cost benefits and capabilities relative to conventional systems such as deep-brain stimulation (DBS) [9,16–22].

Figure 1*d* illustrates how novel adaptive closed-loop systems are addressing the shortcomings of standard closed-loop systems by incorporating a more complex system architecture with heavy reliance on external computational power to manage control algorithm and feedback signal optimization [22,36]. These system models that can still be identified as *cloud/expert-in-the-loop* or *phone/patient-in-the-loop*, require continuous or, if pre-identified, regular data telemetry for their machine learning models to be trained on the cloud or smartphone. Many works can be identically or partially identified as offloading resource intensive tasks externally, with reliance on distributed software at the heart of these closed-loop systems [23–32,37]. To list a few examples, the role of a processing unit, in innovations such as [32], is limited to the controller of system operation. Another example is outsourcing training, improvement, and optimization of on-chip models to the cloud by regular and continuous transfer of device database [30]. It should be noted that some of the aforementioned methods may offer active adjustments to the system on-the-fly, but none enable on-chip training and re-training, which should be at the core of a successful and highly personalized seizure forecasting system. An idea that offers active learning on device was proposed in [38]. In that work, the authors used several deterministic feature extraction techniques and relied on an ideal detection, which is a strong assumption to make, if not practical.

Our alternative is AURA, shown in figure 1*e*. The combination of an on-chip and actively evolving forecasting model with a *brain loop-recorder* and a dependable detection model provides an adaptive and personalized system with innovative potential for closed-loop neuromodulation. Our early empirical evaluation on seizure forecasting shows how self-supervision can mitigate the need for continuous and demanding data telemetry to and from external entities from the loop. The AURA platform still allows for on-demand data telemetry for system diagnostics and performance monitoring.

To test AURA in a potentially practical challenge that is susceptible to data drift, we have identified seizure forecasting as an optimal use-case, illustrated in figure 1. Firstly, the early prediction of a seizure allows for preventative action to take place, such as closed-form feedback via neurostimulation. Secondly, early warning signs manifest in different ways across patients, such as varying brain-wave patterns, and adapting a network to learn the neural signature of a patient could lead to better prediction results. Finally, the largest publicly available labelled scalp electroencephalography (EEG) seizure dataset lacks sufficient information prior to the onset of seizures [6]. The absence of such data means seizure forecasting has been a challenging task for deep learning models, and there is a need to develop techniques that can train and tune models from unlabelled (or indirectly labelled) datasets as well. AURA is perfectly poised to fill this void.

## 2. Background

### 2.1. Seizure forecasting

It was long thought that epileptic seizures were abrupt events that would materialize without prior warning [39], but the advent of long-term EEG recordings changed this. In the early 1970s, it was shown that seizures could develop over long time scales [40] which pointed to seizure forecasting potentially being within reach. Since then, evidence has amassed to show that seizures are often preceded by detectable changes in brain activity [41–43], where, for example, a significant increase in blood flow occurs within the epileptic hippocampus prior to temporal lobe epilepsy [44,45].

An early seizure warning system could improve patient quality of life by triggering pre-emptive administration of therapies, such as anti-epilepsy medication or electrical stimulation [46], which could avert impending seizures and minimize risk of injury. The minimum time interval between an alarm being raised and the occurrence of the seizure while still rendering an intervention to be possible is known as the seizure prediction horizon (SPH).

### 2.2. Detection is easier than prediction

Detecting a seizure at the time of or immediately after onset has had far more success over forecasting seizures in advance [47]. Several machine learning techniques have been used to augment neurologist readings, leading to faster conclusions while maintaining specialist-level EEG-reading performance for the identification of seizures as they happen [48–51]. Unfortunately, seizure aversion is no longer an option when relying on detection mechanisms alone. There are several challenges that face early onset seizure prediction:

— onset patterns vary greatly between patients [52];
— pre-ictal recordings in one patient can be very similar to non-seizure recordings in another patient [53]; and
— the transition to a pre-ictal state consists of subtle changes that can easily go undetected [54].

The first two challenges highlight the difficulty of developing techniques that generalize across patients. These can be addressed by integrating precision medicine techniques that adapt predictive models tailored to the needs of individual patients.

The third point relates to the challenges of function estimation in temporal data analysis. In deep learning, the goal is to learn a function that maps an input (EEG signals) to an output (whether a seizure will occur after the SPH). The output is treated as a random variable. As the SPH increases, the signal of measurable bio-markers is reduced causing the variance of the output to increase. An intuitive interpretation is that the unpredictable component of the output dominates the predictable component as the time window of forecasting is increased. Reducing the variance of deep learning models can generally be achieved by gathering more data. Unsurprisingly, unlabelled data are far more accessible than labelled data.

### 2.3. The bulk of medical data is unlabelled

Patient-specific models and training with large datasets are somewhat conflicting notions. Individual patients cannot contribute the same scale of data as a whole population of patients. While the availability of long-term EEG recordings has renewed interest in designing patient-specific forecasting algorithms [55–57], these algorithms still underperform when compared to seizure detection.

The world’s largest public seizure database, the Temple University Hospital (TUH) seizure corpus, contains EEG recordings of over 4000 patients, and is commonly used for testing and validating the performance of seizure detection systems that achieve close to expert clinician performance [6,48]. The recordings commence between 5 and 35 min pre-ictally which limits the length of the SPH. While there is a plethora of unlabelled data, the lack of formally trained EEG readers available to provide precise temporal annotations, with confirmatory secondary readings, means this huge source of data cannot be directly used in supervised learning methods. The bulk of medical data remains unlabelled and underused.

This challenge can be addressed by relying on high-performance seizure detection models to annotate these data for us. Weak supervision has previously been applied by obtaining inaccurate labels from a mix of experts and novices for real-time seizure detection [58]. The study by Saab *et al.* shows that deep learning models trained using a large body of imperfect labels can outperform a small dataset of perfect, error-checked annotations [58]. The use of weak supervision has become prevalent in modern deep learning to broaden the pool of usable data [59].

Our approach can be distinguished as we wholly do away with manual annotations, and instead use a detection model shown to perform similarly to neurologists [48,60]. These machine-generated detection labels are then used as targets for the prediction model. Some of the detected seizures may be misclassified (and thus, weak labels for prediction), which raises concerns that noisy labels could mislead the prediction system [61].

Fortunately, in the real world, the fact that temporal data are inherently correlated is extremely useful. A seizure that is misclassified at its onset (real-time detection) is unlikely to be successfully predicted pre-ictally (forecasting), as the unpredictability of seizures generally increases with a longer SPH. Therefore, such errors are treated as inevitable; the larger amount of correctly classified data is instead used to offset potential performance degradation from noisy labels. Training a prediction system using approximate labels derived from a detection system makes better use of temporal correlations in the real world [61–63].

### 2.4. Learning patient-specific patterns

Seizures may be regarded to follow patient-specific cyclic patterns. This has been long observed since 1939, when Griffiths and Fox observed that some patients experience seizures at certain times of the day, while others followed monthly cycles [64]. Confounding variables also contribute to the variance between patients, including medication, stress, circadian effects, and hormonal effects, among others [65–68]. Circadian (days) and multidien (multi-days) seizure cycles have also recently been studied with patient self-reported diaries and retrospectively on some long-term intracranial EEG data, which show peaks in seizure cycles as long as 30 days apart [69–72]. Whether the observed cycles are valid and whether they can be linked to triggers such as missed medication, mental and emotional states, the menstrual cycle, and the duration and severity of seizures require objective and prospective studies [73]. It is known that mammalian physiology and behaviour are widely influenced by light and other environmental factors, which means circadian or multidien studies require well-developed protocols before the study is conducted [74].

Personalization in bio-marker discovery and response to therapies continue to demonstrate its profound significance in the field, but it is poorly understood [75,76]. With respect to seizures, studies in drug-resistant focal epilepsy show multi-dimensional individualization not only across different patients, but also between seizures in one patient [76,77]. Personalization is also reported in studies concerning seizure cycles, while more should be done to properly address possibilities for seizure triggers, and accommodate protocols associated with circadian studies [25,72,78–81].

This poses a challenge in developing models that generalize across populations. The present approach to designing personalized forecasting models relies on individualized labelled data, which demands precise temporal annotations for each future time window during training. While this may be feasible for small-scale datasets, it is not a long-term tenable solution for challenging tasks, such as seizure forecasting, or for patient-specific tuning with a large population of patients.

### 2.5. AURA

**Figure 2.**
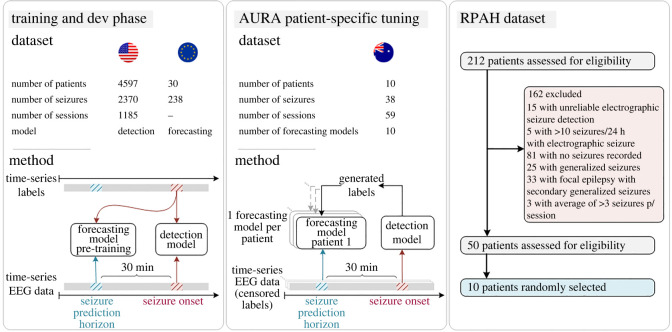
AURA methodology as applied to seizure prediction. The detection model is trained using a US dataset, and the prediction model is pre-trained with an EU dataset. The AURA patient-specific tuning phase assigns a replica of the pre-trained prediction model to each patient from the Australian dataset, and all ground-truth labels are censored. The global detection model generates labels in real time which are used to train the 10 patient-specific prediction models across 59 sessions of EEG recordings. Each patient has a personalized forecasting model that has been tuned to their pre-ictal signatures. To provide a measure of forecasting performance, the labels are declassified and compared to the predictions generated during the AURA patient-specific tuning process.

This work proposes AURA, an adaptive, unlabelled, real-time and approximate approach to online learning, and its performance is demonstrated on patient-specific seizure forecasting. An overview of AURA as applied to seizure forecasting using several datasets procured across three different continents is illustrated in figure 2 (described in further detail in Methods). AURA consists of a real-time detection network and a forecasting network. The detection network classifies the onset of seizures in real time with acceptable accuracy, and the forecasting network uses the output of the detection network as labels. This allows training to take place with the plethora of unlabelled data that is available, and pre-trained forecasting networks can be retrained to adapt to individual patients in order to learn patient-specific pre-ictal signatures.

This approach is a narrow form of semi-supervised learning, where a pre-trained detection model achieves acceptable performance on a modestly sized dataset, while the forecasting network is updated using initially unlabelled data. Although inaccurately labelled data may deceive the forecasting network, AURA is implemented with the inductive prior that the forecasting network is unlikely to predict what fails detection. The irreducible error of the detection model must therefore be less than that of the prediction model, which is a reasonable assumption for temporally correlated prediction and detection. Performance degradation is compensated for by expanding the pool of usable data by the forecasting network for training.

We perform a pseudo-prospective study using AURA across 10 patients. The detection network is trained on patients from a US-based hospital, which generates labels during the AURA online learning process on a sample of patients from the Royal Prince Alfred Hospital (RPAH) in Sydney, Australia. Datasets from different hospitals are used to demonstrate that AURA leads to a performance improvement, even when generalizing across patients from different continents. The AURA-trained forecasting networks show an average improvement in relative sensitivity by 14.30%, and a reduction in false alarms by 19.61%. This early demonstration of AURA on real-world patients in a pseudo-prospective trial acts as a feasibility study towards its use on a larger cohort of patients. A high-level overview of the AURA training process is depicted in figure 2.

## 3. Datasets

The seizure detection model is trained on the TUH seizure corpus [82] from the USA, while the prediction model is pre-trained using the European EPILEPSIAE dataset [83]. The AURA self-learning process is applied to the Australian test set from the RPAH where all human-annotated labels have been censored [48], and each patient starts with the same pre-trained prediction model that adapts over the course of their multiple monitoring sessions. Upon completion of all sessions, the sequence of predictions generated by the forecasting network is compared to the uncensored ground truth to provide a performance measure of sensitivity and the number of false alarms. It is important to note that each prediction for a given time window takes place 30 min prior to the detection network generating a label that corresponds to the same window. This ensures our metric for performance using any given sample is reported before the forecasting network is updated on the basis of the same sample. This reduces the risk of future time leakage into our measure of performance, and emulates the operation of AURA in practice.

### 3.1. TUH dataset

The TUH dataset [82] is the world’s largest open EEG database for seizure research. It includes 592 patients in the training dataset and 50 patients in the test set. Due to the lengthy sessions, much of the recordings that do not include seizure onset have been removed. The temporal discontinuity of the data and the lack of sufficient pre-ictal content mean the TUH dataset is not suitable for use with AURA, and would not be representative of real-world usage. However, it is an ideal dataset for training the seizure detection model, despite the seizure and background information imbalance. The details are shown in table 1, in which the total seizure and background duration in the Train/Dev datasets are 46.7 h and 752.3 h, respectively. Public access to TUH dataset is possible via online registration and application for access.

**Table 1.** Summary of TUH dataset.

attribute	train	dev
files	4597	1013
sessions	1185	238
patients	592	50
files with seizures	867	280
sessions with seizures	343	104
patients with seizures	202	40
number of seizures	2370	673
background duration (hours)	705.6	154.1
seizure duration (hours)	46.7	16.2
total duration (hours)	752.3	170.3

Note: Training the detection model follows the same procedure as in [48]. The TUH seizure corpus provides dedicated validation and test sets, although the labels from the test set are unreleased. Therefore, the default validation set was instead used as a held out test set for performance benchmarking of the detection model, while the train set was randomly split with a ratio of 80–20 to create our own validation subset.

### 3.2. EPILEPSIAE dataset

The EPILEPSIAE dataset is the largest continuous EEG database in Europe which contains a total of 275 patients [83], among which, scalp-EEG recordings taken from 30 patients are made publicly available. Although this number is much fewer than the TUH dataset, the recordings of all patients are significantly longer in duration, in the range [92.9,266.4] h. A summary of patient details is provided in table 2 with a total of 238 seizures across a 4604 h recording duration. This dataset is used to pre-train the seizure prediction model. Public access to the EPILEPSIAE dataset is possible and requires payment, registration and application for access.

**Table 2.** Summary of EPILEPSIAE scalp-EEG dataset.

patient	gender	age range	SN	seizure foci	RD (h)	mean SD (s)	range SD (s)
1	M	36 − 40	11	central and parietal	164.7	78.3	[54.5, 162.0]
2	F	46 − 50	8	temporal	177.4	52.9	[4.3, 71.3]
3	M	40 − 45	8	temporal	143.3	58.6	[34.3, 102.8]
4	F	66 − 70	5	temporal	167.8	121.0	[84.3, 166.1]
5	F	50 − 55	8	frontal and temporal	266.4	92.3	[11.3, 379.1]
6	M	60 − 65	8	temporal	135.4	50.2	[0.0, 105.1]
7	M	36 − 40	5	temporal	118.1	46.7	[29.3, 79.6]
8	M	26 − 30	22	frontal	115.6	20.1	[0.9, 96.3]
9	M	46 − 50	6	temporal	94.0	71.4	[65.9, 83.8]
10	M	40 − 45	11	frontal and temporal	138.0	39.9	[0.0, 68.1]
11	M	46 − 50	14	central and temporal	138.1	63.6	[19.4, 100.4]
12	M	26 − 30	9	temporal	159.7	41.4	[31.2, 60.8]
13	M	46 − 50	8	frontal and temporal	158.1	91.0	[55.0, 125.5]
14	F	60 − 65	6	temporal	162.2	124.0	[83.0, 174.1]
15	F	40 − 45	5	temporal	118.7	64.9	[7.6, 144.0]
16	F	10 − 15	6	temporal	92.9	56.1	[2.5, 99.0]
17	F	16 − 20	9	temporal	159.1	55.8	[33.3, 76.5]
18	M	46 − 50	7	temporal	178.2	41.7	[22.7, 69.4]
19	M	30 − 35	22	temporal	161.1	65.2	[43.1, 96.5]
20	M	46 − 50	7	temporal	164.6	59.5	[19.1, 119.8]
21	F	30 − 35	8	occipital	159.4	51.9	[9.3, 118.9]
22	M	36 − 40	7	parietal	137.9	95.7	[55.5, 145.1]
23	M	50 − 55	9	temporal	237.5	56.1	[18.8, 88.0]
24	F	50 − 55	10	temporal	94.4	72.9	[49.6, 122.8]
25	M	40 − 45	8	central	159.7	329.3	[73.8, 909.2]
26	M	10 − 15	9	temporal	159.0	62.7	[38.0, 104.5]
27	M	56 − 60	9	temporal	159.5	95.3	[43.9, 331.2]
28	F	30 − 35	9	temporal and parietal	162.3	86.2	[59.4, 216.9]
29	M	50 − 55	10	temporal	161.1	180.7	[62.1, 270.0]
30	F	16 − 20	12	temporal	159.8	75.2	[10.0, 151.9]
total	—	—	238	—	4604	75.9	[0.0, 909.2]

M, male; F, female; SN, number of seizures; RD, recording duration; mean SD, mean of seizure duration; range SD, seizure duration range.

### 3.3. RPAH dataset

**Figure 3.**
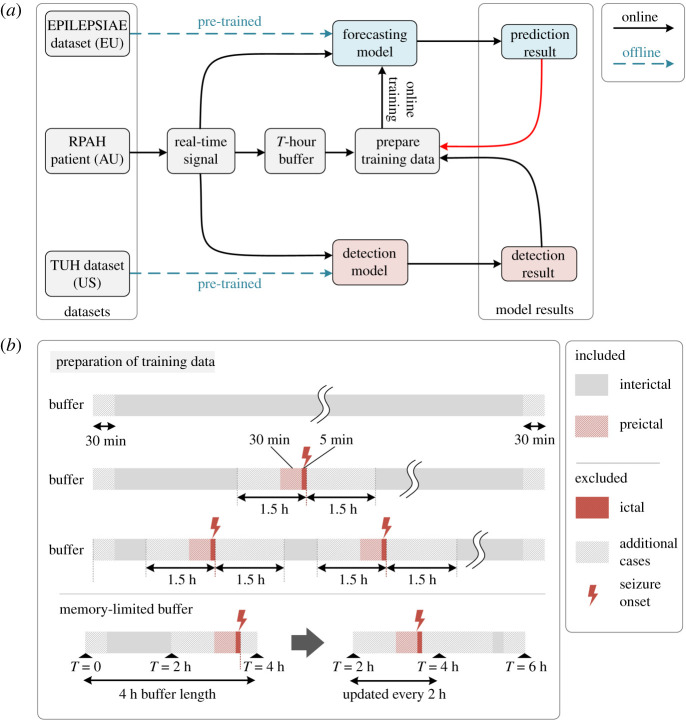
Online learning procedure. (*a*) The prediction and detection models are initially pre-trained offline. The EEG recording from a given patient from the RPAH dataset is streamed as a real-time signal. It is used to generate a real-time detection (detection result) and a 30 min forecast (prediction result). The detection result (i) determines whether the sample is used to train the prediction model and (ii) is used as the target label for the prediction model outcome from 30 min prior. The red arrow between ‘prediction results → prepare training data’ indicates that prediction results can be used to govern the training of the prediction model; e.g. if the prediction results are aligned with the detection results, there is no need to update the prediction model. (*b*) Training data preparation. If the detection result indicates an inter-ictal or pre-ictal (within 30 min of onset) reading, online training of the prediction model is enabled. If the reading is ictal (within 5 min of the seizure onset), post-ictal (within 1.5 h after seizure onset), online training is disabled as the signal is considered ‘contaminated’. In the 4 h buffer, the first and final 30 min of the buffer are not used for training. The reason is because prediction labels do not exist for the first 30 min, and in the final 30 min, prediction labels from the seizure prediction horizon (30 min) fall outside the range of the buffer. To ensure forecasted seizures in the final 30 min are still accounted for, incoming data sequentially replace the oldest data in the buffer, emulating the function of a loop recorder. The minimum usable buffer size must account for the 30 min pre-ictal duration, the 1.5 h gap between ictal and inter-ictal period, 30 min at either end of the buffer, and the seizure duration itself. Our experiments use a 4 h buffer length.

Our selection procedure is detailed in figure 2. There are a total of 212 adult patients with surface EEG recordings at the RPAH (Sydney, Australia), where 192 patients have reliable EEG recordings (excluding 15 patients that are lacking full electrode information, and another five patients that experience more than 10 seizures/24 h). Among the remaining potential subjects, 111 patients have seizures recorded. The pool of patients is narrowed to those with focal epilepsy who: (i) do not experience generalized or secondary generalized seizures, as the initial source of each seizure varies, (ii) experience at most three seizures per day on average to provide sufficient inter-ictal training time (figure 3*a*) and (iii) have at minimum three sessions (approx. 3 days) recorded. Fifty patients are assessed for eligibility; among these patients, 10 patients are used as subject-under-test for real-time AURA training across a total duration of 949.9 h. Detailed information for each patient is provided in table 3. Each patient starts with an identical pre-trained forecasting model, which is adapted during the AURA process.

**Table 3.** Summary of RPAH selected dataset.

patient	gender	age range	seizure no.	session no.	RD (h)	mean SD (s)	range SD (s)	seizure type	seizure foci	secondary generalized
1	F	20 − 25	3	7	116.3	50.5	[47.3, 53.6]	focal	LO	N
2	F	50 − 55	4	6	95.1	70.4	[29.6, 94.0]	focal	RF	N
3	M	40 − 45	1	4	70.6	118.3	[118.3, 118.3]	focal	LT	N
4	F	46 − 50	3	7	158.8	76.7	[65.8, 92.8]	focal	LP-O	N
5	F	30 − 35	7	5	53.7	61.1	[36.2, 91.3]	focal	LT	N
6	M	46 − 50	6	8	94.3	48.0	[36.5, 56.4]	focal	RT	N
7	M	66 − 70	7	5	55.9	55.6	[46.7, 67.7]	focal	LT	N
8	M	46 − 50	2	4	46.9	64.7	[58.2, 71.2]	focal	RT	N
9	M	60 − 65	2	8	162.6	62.1	[59.4, 64.8]	focal	RT	N
10	F	30 − 35	3	5	95.2	219.3	[138.0, 363.7]	focal	RT	N
total	—	—	38	59	949.4	73.6	[29.6, 363.7]	—	—	—

M, male; F, female; SN, number of seizures; RD, recording duration; mean SD, mean of seizure duration; range SD, range of seizure duration; secondary generalized, seizures that initiate focally and end with bilateral motor activity. They are often clinically classified as focal seizures. Seizure foci: LO, left occipital; RF, right frontal; LT, left temporal; RT, right temporal; LP-O, left parieto-occipital.

This study on the RPAH clinical data is approved by the local Research Ethics Committee. Ethics approval no. *X*19-0323-2019/STE16040 on *Validating epileptic seizure detection, prediction and classification algorithms* approved on 19 September 2019 by the NSW Local Health District for implementation at the Comprehensive Epilepsy Services, Department of Neurology, The Royal Prince Alfred Hospital (RPAH). The RPAH data are not openly available to the public.

## 4. Methods

### 4.1. Seizure prediction using AURA online learning

The online learning process using real-time streamed EEG data is shown in figure 3. The surface EEG signal of a given patient is read into the buffer in figure 3*a* once per second, where it is stored until it reaches *T* hours. In our implementation, our buffer was limited to *T* = 4 h. Once the total duration of the recording exceeds *T*, new data are loaded into the buffer while earlier data are sequentially cleared which ensures positive forecasts in the final 30 min of the buffer are accounted for. Concurrently, the two models generate detection and prediction outputs for batches of 12 s and 30 s signals, respectively. Once the buffer reaches *T* hours, the training data for the prediction model are prepared based on the detection result (weak label), as illustrated in figure 3*b*. EEG signals associated with inter-ictal and pre-ictal (up to 30 min) data are included in the online training process. Ictal and post-ictal (up to 1.5 h) data are excluded from the online learning process, as generating forecasts at seizure onset would be based on contaminated signals. The first 30 min of EEG recordings in the buffer is also excluded as there is no guarantee the patient is not in a post-ictal state, and also due to the absence of forecasted labels in the first 30 min. The final 30 min is excluded as there is no knowledge of the state of the patient, e.g. a seizure may occur right after the buffer. This ‘right-censored’ recording is accounted for in subsequent steps once the buffer is updated over time, and the data become uncensored by shifting along the buffer’s storage. Once the system has flagged a data sample for inclusion in the online training process, the weak label from the detection model is compared to the prediction result using a negative log-likelihood loss function, followed by a gradient calculation step via the backpropagation algorithm.

#### 4.1.1. Real-time signal pre-processing

Once the patient’s real-time signal accumulates to a 12 s window, independent component analysis (ICA) [84] and short-time Fourier transform (STFT) are applied to the EEG signal before being passed to the pre-trained seizure detection model. ICA is applied to decompose the signal into several statistically independent components by using the blind source separation (BSS) [85] approach. Equation (4.1) shows the working principle for the BSS, where $T\in R^{I_{t}\times I_{e}}$ represents the multi-channel EEG signals, *I*_*t*_, *I*_*e*_ indicate the number of samples over time, and the number of electrodes, respectively. After the decomposition, $M\in R^{I_{t}\times R}$ and $A\in R^{I_{e}\times R}$ contain decomposed signal temporal information and topographic weight map, respectively, where *R* is the estimation of source number.

4.1 $$T\approx MA^{⊤}.$$

The electro-oculography (EOG) channel records eye movement information, and is physically proximate to the ‘FP1’ and ‘FP2’ EEG channels. In some particular cases, EOG may be indicative of a seizure. However, in the majority of cases, EEG has been shown to be susceptible to contamination by EOG signals [86], and a significant body of work has shown that removing EOG artefacts in EEG improves the performance of seizure identification [87,88]. Thus, ICA is used for the purpose of removing the EOG artefact, which is performed using the MNE Python package [89]. Independent components of noise-prone EEG channels above a defined Pearson correlation [90] threshold with the EOG channel are removed.

STFT [91] is then applied to the clean EEG waveform with a 250 sample window (1 s) length and 50% overlap. The DC component of the transform is also removed as it is known to have no relation to seizure occurrences. The same technique is used on the real-time stream of EEG data for the prediction model, but using 30 s windows instead. Equation (4.2) shows the Fourier transform formula that calculates frequency domain information for the input signal, where *m* represents the window length and *n* for the *n*th sample. The magnitude is calculated by absolute value after the STFT, which is shown in equation (4.3). STFT is implemented in Python 3.8 using the STFT package.

4.2 $$X(m,\omega )=\sum_{n=0}^{\mathrm{end}}x[n]\omega [n-m]\;e^{-j\omega n}$$

and

4.3 M(m,ω)=|X(m,ω)|.

#### 4.1.2. Seizure detection and prediction pre-trained models

All pre-training takes place offline, where the seizure detection model is trained using the TUH dataset (see §3.1) and the prediction model using the EPILEPSIAE dataset (see §3.2). The detection and prediction models both consist of convolutional long short-term memory modules [92] combined with a pair of fully connected layers, based on our previous work on seizure detection [48]. A summary of the architecture is provided in table 4.

**Table 4.** Network architecture.

layer	type	parameters
1	ConvLSTM	*k* = (*n* × 2 × 3), *f* = 16, *s* = 1
2	ConvLSTM	*k* = (16 × 1 × 3), *f* = 32, *s* = (1 × 2)
3	ConvLSTM	*k* = (32 × 1 × 3), *f* = 64, *s* = (1 × 2)
4	FC	*m* = 256, sigmoid
5	FC	*m* = 2, sigmoid

*k*, kernel dimensions; *n*, input channel dimensions; *f*, number of filters; *s*, stride; FC, fully connected layer; *m*, number of neurons.

The pre-trained detection and prediction model is implemented in Python 3.6 using Keras 2.0 and TensorFlow 1.4.0. To avoid overfitting, all fully connected layers use a dropout rate of 0.5, and early-stopping techniques are applied (patience of 20 epochs based on the combined training and validation set loss) to terminate the training process. The Adam optimizer [93] is used during the training, and batch size and the learning rate are set as 32 and 5 × 10^−4^, respectively. It took approximately one day with an NVIDIA V100 GPU to train the model.

#### 4.1.3. Buffering

In our system, the buffer has a size of 4 h of loop EEG recording. Specifically, every 2 h, new EEG signals are added to the buffer and old signals are flushed in a first-in-first-out fashion. The buffer also contains timestamps of the EEG signals, which are used to prepare the training data. Note that the size of the buffer (4 h in this work) and the frequency of updating the buffer (2 h in this work) are subject to the available computation and memory resources. In other words, the online training process that uses the buffered data must finish before the next buffer update. This process is depicted in the illustration of the memory-limited buffer in figure 3*b*. The buffer consists of 4 h of EEG data, split into a pair of 2 h buffers. The second buffer is updated at 2 h intervals. The first 2 h buffer accumulates and maintains the real-time incoming stream of data. Forecasting and detection are performed in real time on the stream of incoming data, and the total buffer is therefore used for fully automated annotation and training purposes. The minimum length of the buffer, which represents the interictal duration used in active training of the prediction model, must be consistent with the minimum length of the time considered as interictal data can be defined. Ideally, a longer buffer duration is preferred to account for long inter-ictal periods, but in this case, a 4 h buffer achieved a good balance between the size of the training data and buffer capacity (which is 300 MB on all 19-electrode EEG) for seizure prediction.

#### 4.1.4. Preparation of training data

The forecasting model’s online training data preparation process commences once the buffer reaches *T* hours (*T* = 4 for our experiments). The feedforward detection model labels all suspicious instances of seizure onset. There are three possible cases that may arise in the 4 h buffer. *Case 1*: if no seizure is labelled in the *T*-hour buffer duration, the entire duration of the buffer will be labelled inter-ictal (negative sample), other than the first and final 30 min. *Case 2*: if only one seizure is labelled in the *T*-hour buffer, the 30 min preceding the seizure is labelled pre-ictal (positive sample), and any information that falls 1.5 h away from the seizure onset within the buffer is labelled inter-ictal (negative sample). *Case 3*: if more than one seizure is marked during the *T*-hour buffer, as before, a positive (pre-ictal) label is assigned 30 min preceding the seizure, and a negative (inter-ictal) label is assigned at least 1.5 h away from all seizures. Once the entire buffer consists of labelled EEG information, only those with inter-ictal and pre-ictal labels are further pre-processed using 30 s time windows and STFT as described in §4.1.1.

To ensure the quality of training data, we monitor the signal’s amplitude across all channels and drop segments of the signal (and their corresponding labels) that have overflow or missing data. We also applied ICA to remove artefacts caused by eye movements.

#### 4.1.5. Online training

During the real-time prediction phase, the prediction model is continuously updated based on the results of the detection labels. An adaptive batch size (varied from 5 to 32) is used, with the exact size based on the number of training samples, noting that even for a fixed buffer size, the criterion in §4.1.4 alters the number of samples flagged for training based on the occurrence and timing of seizure alarms. The Adam optimizer [93] is used with a learning rate of 5 × 10^−8^. The purpose of using a small learning rate and batch size is to avoid overfitting caused by the small amount of training data within the *T*-hour buffer.

#### 4.1.6. Post-processing

**Figure 4.**
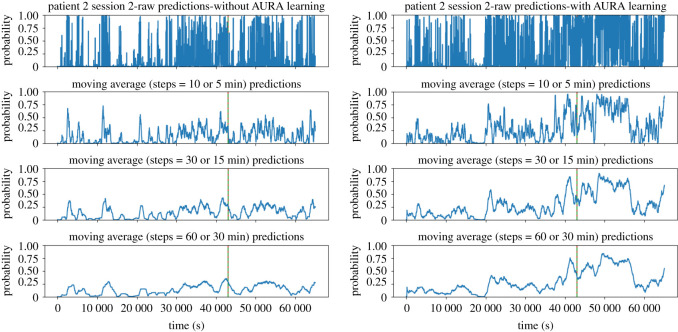
Real-time prediction comparison of a sample patient session without AURA learning (left panels) and with AURA learning (right panels). The dashed vertical red line represents the ground truth seizure onset time, and the solid green line is the weak label generated by the seizure detection model. In the example, these two lines overlap which means the detection model correctly classified the actual seizure onset. The peak that precedes the seizure event corresponds to the prediction system forecasting a high probability that a seizure will occur within the next hour. The peak in AURA learning is far more distinguishable than that without AURA learning. The sample is taken from the second session of EEG recordings, resulting in different probabilities at *t* = 0 s as the model has undergone one session of AURA training.

During the online training process, raw prediction results are further post-processed by calculating the moving average value during a given period, as shown in figure 4. In our experiments, we use a window of 30 min. A patient-specific threshold is applied, where if exceeded, an alarm is raised, predicting the patient will experience a seizure within the next hour.

#### 4.1.7. Performance metrics

The sensitivity and number of false alarms per 24 h are used to evaluate the performance of AURA in real-time seizure prediction for clinical usage. When the prediction results are greater than a specific patient-based threshold, one alarm is raised. The thresholds are chosen for each patient to achieve a balance between sensitivity and false alarm. An alarm is considered correct if it is raised within 1 h of the actual seizure onset. Where multiple incorrect alarms occur within the same hour, they are collectively regarded as one false alarm. Note that during the training process, we define pre-ictal window of 30 min as it has empirically proven to be effective in training a seizure prediction system [94]. This temporal discrepancy arises because the timing of pre-ictal bio-marker onset remains an open question. The sensitivity of the prediction network is calculated by finding the number of correctly predicted seizures over the total number of seizures. The total number of false alarms over all recording sessions is normalized to calculate the number of false alarms per 24 h. The time-in-warning is also calculated to show the amount of time each patient is on high alert, and is calculated by dividing the total time spent in a warning state following an alarm (TP + FP) by the total recording time.

The prediction performance score is also calculated based on the sensitivity and false alarm, inspired by the score formula proposed in the Neureka 2020 Epilepsy Challenge [95]:

4.4 $$\text{score}=\frac{1}{2}\left(\text{SENS}-\frac{1}{24}\times \text{FA/24 h}+1\right).$$

As the predictor alarm is considered to last for an hour, the upper bound on false alarms per 24 h is 24. The coefficients of the score formula are selected to fit the score within a range of 0 to 1. The Wilcoxon signed-rank statistic is used to evaluate the performance of the AURA system compared with the baseline. To account for both sensitivity and the number of false alarms, the *p*-value is calculated based on the patients’ prediction performance score to indicate the significance of performance improvement. The statistical significance threshold was set to 0.01.

## 5. Results

The following pseudo-prospective tests are applied to the RPAH patients:

— *Detection*: The detection model is pre-trained on the TUH dataset and is pseudo-prospectively inference-only real-time tested on the RPAH dataset.
— *Offline-learning*: The prediction model is pre-trained on the EPILEPSIAE dataset and is pseudo-prospectively inference-only real-time tested on the RPAH dataset.
— *Online-learning with AURA*: The prediction model is pre-trained on the EPILEPSIAE dataset and is trained online using the AURA system, using labels that are simultaneously generated by the pre-trained seizure detection model (teacher model), and pseudo-prospectively inference-only real-time tested on the RPAH dataset.

The recordings of each patient from the RPAH dataset are pseudo-prospectively tested on the prediction model with AURA learning, by streaming their scalp-EEG readings into our system in real time. Additionally, the same data are used on the pre-trained prediction model (on the EPILEPSIAE dataset) without AURA learning to identify the effectiveness of the proposed framework. As shown in table 5, all 10 patients show varying degrees of improvement. Patients 1, 2 and 5 have significant improvement, as their sensitivity increases by 33.34%, 25.00% and 14.28%, respectively, while the numbers of FA/24 h decrease by 0.62, 2.01, 0.90, respectively. The result for patient 3 is quite promising as the sensitivity is maintained at 100.00% with approximately 2 FA/24 h with AURA learning. The probability of the null hypothesis, that AURA does not improve the predictive performance score of the forecasting model without AURA, was calculated to be *p* = 0.003, which is smaller than 0.01. The average result across patients without AURA is 55.26% sensitivity, 8.21 FA/24 h, and across patients with AURA is 63.16% sensitivity, and 6.60 FA/24 h. These results are quite promising considering these are non-patient-specific continental generalized tests without human labelling. Other patients 4, 6, 7, 8, 9, 10 show varying levels of FA/24 h, but upon AURA training, there is a consistent decrease of both FA/24 h and time warning, for a constant sensitivity.

**Table 5.** Continental generalization pseudo-prospective results comparison.

prediction	offline learning				online learning with AURA				detection performance		
patient ID	sensitivity (%)	FA/24 h	TW	score	sensitivity (%)	FA/24 h	TW	score	sensitivity (%)	FP/24 h	TW
focal-NSG											
1	—	5.36	0.23	0.55	66.67	**4.74**	0.21	0.73	—	5.98	0.04
2	50	14.88	0.64	0.44	75	**12.87**	0.57	0.61	75	1.51	0.45
3	100	2.72	0.13	0.94	100	**2.04**	0.10	0.96	100	3.30	0.78
4	67.67	4.38	0.20	0.74	67.67	**3.93**	0.18	0.75	100	1.96	0.17
5	42.86	4.92	0.26	0.61	57.14	**4.02**	0.24	0.70	71.43	7.60	1.40
6	—	8.14	0.36	0.50	—	**6.87**	0.31	0.52	83.33	3.05	0.75
7	71.43	15.03	0.72	0.54	71.43	**12.88**	0.63	0.59	71.43	5.02	0.84
8	50	2.56	0.13	0.70	50	**1.54**	0.116	0.72	100	4.50	0.51
9	50	7.97	0.34	0.58	50	**4.58**	0.20	0.65	50	4.43	0.06
10	100	16.64	0.72	0.65	100	**13.87**	0.61	0.71	66.67	7.06	1.25
total	55.26	8.21	0.36	0.61	63.16	**6.60**	0.30	0.68 (p<0.01)	73.68	4.17	0.50

FA/24 h (seizure prediction): false alarms per 24 h, where multiple false alarms occurring within the same hour are treated as one false alarm. FP/24 h (seizure detection): false positives per 24 h, where false positives are counted for every 12 s segment, and multiple false positives within 1 min period are considered as one false positive. TW: time warning is calculated by total alarm time over the total recording time (for the prediction the alarm raise for one hour, while for detection alarm raise for 12 s). Score: the score is calculated based on the formula explained in §4.1.7. The *p*-value is between the 10 patients' scores without AURA and with AURA. Offline learning: the prediction model is pre-trained on the EPILEPSIAE dataset and is pseudo-prospectively tested using inference-only on the RPAH dataset. Online learning with AURA: the prediction model is pre-trained on the EPILEPSIAE dataset and AURA is applied to perform patient-based online training. At the same time, pseudo-prospective testing is performed on the RPAH dataset. Detection performance: the detection model is pre-trained on the TUH dataset and is pseudo-prospectively tested using inference-only on the RPAH dataset.

Figure 4 shows a sample taken from the second session of patient 2 experiencing focal epilepsy. The dashed vertical red line shows the ground truth seizure onset time, and the solid green line represents the weak label generated by the seizure detection model. In this case, the detection model correctly labels the actual seizure onset such that they are almost perfectly overlaid. The first row shows the raw prediction results, and the subsequent rows show a moving average of 5, 15, 30 min results, respectively. From the graph, we can see that without AURA, the inference-only test shows a very low probability of seizure occurrence, while with the AURA training process, the model becomes more sensitive to seizures by showing a higher probability of an alarm being raised prior to seizure onset. This is achieved concurrently with reducing the number of FA/24 h for each patient.

## 6. Discussion

In this study, we present the AURA online learning system that uses patient-specific semi-supervision with a pair of temporally correlated tasks. On our early assessment of 10 patients, we demonstrate that it consistently improves (or maintains) patient-specific seizure prediction results in terms of both sensitivity and the number of false alarms. Based on the average of 10 patients’ results, the real-time seizure detection model outperforms the prediction model, which reaches an average of 73.68% sensitivity, 4.17 FA/24 h, and only 0.50% time warning. Averaging the sensitivity and FA/24 h across all patients shows the prediction model achieves 63.16% sensitivity, 6.60 FA/24 h with AURA online learning, and 55.26% sensitivity, 8.21 FA/24 h with offline learning. With the AURA online learning, the seizure prediction model moves closer to the performance of the detection model. We can see that the seizure prediction result improves across all 10 patients with only several sessions per patient, with an absolute average improvement of sensitivity by 14.30%, and an average reduction of 1.61 FA/24 h. The duration a patient is in time warning also decreased by 16.67%, which has the potential to reduce alarm fatigue in clinical settings once benchmarked on larger patient cohorts.

AURA online learning sits at the intersection of (i) self-supervised learning, (ii) meta-learning and (iii) weak supervision. We achieve our automatic and on-the-fly generated label-base via dependable, generalized and pseudo-prospectively tested inference models of epileptic seizure annotation tools that have been tested in clinical settings [48]. Though the automatically generated labels are not perfect, it has been demonstrated that expanding the label pool and access to greater automatically and cheaply generated labels provides a way to improve an otherwise low-performance initial model [96] (in our case, initial forecasting). When the seizure detector fails, the false training label is passed to the seizure predictor. It may lead to an error during the training process of the seizure predictor, but with a sufficiently large sample of correct labels as with seizure detection, the training process will continue to improve upon a baseline model. The seminal work by Ratner *et al*. [96] asserts that generating perfectly labelled data within constrained resources is often impossible and even generating ‘mostly’ correct labels is expensive. Moreover, one of the goals of this paper is to show that even in light of erroneous labels during detection, the final performance of the forecasting model offers an improvement over a baseline model (baseline model = a model without continual learning). Our results in table 5 provide early evidence that our system is tolerant of label errors with statistical significance. We have additionally already provided performance data of the label-generation mechanism to offer further reader detail on quantitative information about the error rate.

### 6.1. Adapting to patient-specific biomarkers

In 2001, a five-patient clinical study was conducted that suggested epileptic seizures commence after a cascade of electrophysiological events which occur far earlier than clinical onset [97]. Our recent work [98] postulates that slowing inter-ictal activities are a potential biomarker for epileptic seizure prediction. The gradual improvement of results using AURA, to some extent, supports the hypothesis that patient-specific early warning signs are regularly raised before the seizure onset.

The three patients with the largest margin of improvement (1, 2 and 5) interestingly have focal epilepsy with different specific seizure foci: left occipital, right frontal and left temporal, respectively. Additional details are provided in table 3. Thus, for patient-specific online training, if a biomarker exists and the detection model can continuously and correctly generate labels of seizure onsets, the forecasting model can theoretically learn to identify seizure prediction biomarkers for that specific patient.

### 6.2. Dataset generalization

AURA is demonstrated on a different source of dataset from that on which the detection and prediction models are pre-trained, and is representative of different populations and recording practices. There are two instances where the system is required to generalize in our experimental system. Firstly, the detection model is trained on the US-based TUH dataset but must generate labels for the Australian RPAH dataset. Secondly, the prediction model is initialized with pre-trained parameters from the European dataset, but performance is reported on the Australian patients. Both prediction and detection models run in real time simultaneously, and if a seizure occurs, the prediction model will ideally report the warning 30 min before the detection model is exposed to its first sample of seizure onset data. After the detection model detects the seizure onset, the pre-ictal information will be buffered and used in online training of the prediction model. Therefore, the prediction performance is reported 30 min prior to updating the model via backpropagation using that particular information in the calculation of the online training loss. In reporting prediction performance 30 min prior to the network update, we also avoid future time leakage in reporting our results. This reduces the risk of future-time leakage.

The performance of the detection model for each patient is also reported in table 5, where the average performance of detection is better than that of the prediction system with AURA learning. While this result is unsurprising, i.e. AURA is implemented on the basis that the upstream task of detection is easier than the downstream task of prediction, it is interesting to see AURA leads to better forecasting for patients 1 and 3 in both sensitivity and FA/24 h than detection. The detection model evidently does not impose an upper bound on performance, but this counterintuitive result may be attributed to the detection network struggling to generalize. For example, if pre-training of the detection network were to take place on the same RPAH cohort, then the irreducible error of the detection system would likely be less than that of the prediction network, even with AURA learning. This is consistent across all 10 patients, who experience seizure prediction performance that moves closer to that of detection. The patients show varying levels of improvement, and more patient tests and experiments from a wider variety of seizure types must be implemented to instill higher confidence in the proposed system. Moreover, more than one detection model can be integrated into AURA for generating labels. These labelling sources can include: deep learning-based algorithms, deterministic algorithms (e.g. [42]), and commercially available seizure detection software (e.g. Encevis-Episcan [60]). Multiple labelling sources can be combined with the Snorkel method presented in [96].

### 6.3. Towards on-chip online learning for neuromorphic neuromodulation systems

AURA may be the first step towards designing a patient-specific and highly customized low-power neuromorphic neuromodulation system, where sufficient performance for a given patient may justify closed-loop neurostimulation for seizure avoidance. Developing a continuously updated, always-on neuromorphic system that adapts to a patient’s bio-markers requires a low-power, portable learning system that can be ambiently deployed for outpatient care [99,100]. Deep learning, and particularly training models, is notorious for its large energy consumption which arises due to the huge number of parameters in large-scale models [101–105], and corresponds to frequent memory accesses and data movement across a chip and between chips. While our prototypical demonstration of AURA provides much promise in the potential of targeted deep learning models in seizure forecasting, extending usage beyond inpatients requires overcoming the energy and latency bottlenecks of on-chip learning [106–109].

The fields of neuromorphic computing and emerging memory technologies are ushering in new algorithms and architectures that demonstrate how modern deep learning and neuromorphic event-driven systems can be modified to cater for resource-constrained environments. For example, the use of spiking neural networks can reduce the power consumption of equivalent deep learning algorithms by several orders of magnitude [110–114], and in-memory computing architectures that co-locate processing with network parameter storage can achieve similar performance improvements. Although online/transfer learning and real-time variants of backpropagation algorithms are not often used in practice, AURA as applied to at-risk outpatient tuning motivates a compelling use-case for new generation, online techniques for handling deep learning algorithms, tailored to highly customized low-power neuromorphic neuromodulation systems [33,34,115]. Event-driven neuromorphic computing allows tremendous energy efficiency in both training and inference, but the focus has been primarily on inference engines due to its ubiquity in the IoT (Internet of Things). Recently, significant energy reduction in edge-based training has been demonstrated, where an online transfer learning image processing neuromorphic chip is reported to operate on 23.1 mW (23.6 mW) with 0.8 V supply voltage at 20 MHz, while processing 93 k 28×28 images/s (100 k 28×28 images/s) during training (inference) [33]. While the simplicity of the network reported here may not be suitable for complex neurophysiological data processing at this point in time,^1^ a highly customized low-power neuromorphic neuromodulation system is a promising avenue to implement and deliver this paper’s vision beyond power-hungry complex deep learning models.

We envision the potential use of our system as a real-time prediction system with the model learned by the weakly supervised label. Where either the seizure detector or predictor fails, false information is passed to the doctor and patient, and may lead to inappropriate action being taken to handle the seizure. However, non-automated systems are not completely fail-safe, either. The proposed system may act optimally as a decision support system for clinicians to partially mitigate the risks.

### 6.4. Future works

This paper proposes a novel approach to seizure forecasting via online learning by using weak labelling from seizure detection. This is verified by a feasibility study on 10 randomly chosen patients. In our future work, we plan to test more patients from different sources to demonstrate the potential capacity for generalization of our forecasting approach. This core idea may also be verified in other applications from completely different domains, for example, in weather detection and prediction.

## 7. Conclusion

Human labelling of EEG data is an expensive and laborious process. Our approach to weak self-supervised learning using the proposed AURA system shows a potential direction in overcoming the challenges and costs of individualized labelling to deploying patient-specific models that adapt to patient-specific bio-markers. AURA shows robustness to the use of prediction and detection models that are pre-trained on different sources of data, and beyond precision medicine applications in seizure detection, AURA introduces a feasible way to harness the multitude of unlabelled time-series clinical data that remain underused in deep learning. The RPAH dataset is limited in the number of sessions (and therefore, ictal events) per patient, which adds to the difficulty of training AURA, but we successfully demonstrated an improvement for each tested patient despite this constraint all without the use of clinician labels. Although more experiments need to be conducted to verify the efficacy of our proposed system on wider populations and a broader range of seizure types, our early demonstration shows promising utility for real-world clinical utility.

## Data Availability

The RPAH data are not openly available to the public. Australian policy is restricted to clinical data containing sensitive and identifiable information. The approved ethics prevented us from the public release of unidentified data and at this stage only allowed limited research access. The Temple University Hospital (TUH) dataset is freely available publicly, and no payment is required to access the data. These data can be accessed through the link https://isip.piconepress.com/projects/tuh_eeg/html/downloads.shtml after filling the form by providing the institution and email information. The TUH EEG Seizure Corpus (TUSZ) v. 1.5.1 is used in this paper. Access to the EPILEPSIAE dataset requires payment to maintain the data and support the development of the high-quality data: http://www.epilepsy-database.eu/. The source code used in the paper is publicly available at https://github.com/NeuroSyd/Weak_learning_for_seizure_forecasting and has been archived within the Zenodo repository: https://doi.org/10.5281/zenodo.6802533.

## Footnotes

1 See the cheap gradient principle [116].
